# Structural Effects on the Energy Disposal and Atomic Photofragment Alignment for the Photodissociation of Alkyl Iodides at Excitation Wavelengths of 254 and 268 nm

**DOI:** 10.1021/acs.jpca.4c02217

**Published:** 2024-09-19

**Authors:** Javier Cachón, Pedro Recio, David Sorribes, Sonia Marggi Poullain, Luis Rubio-Lago, Luis Bañares

**Affiliations:** † Departamento de Química Física, Facultad de Ciencias Químicas, Universidad Complutense de Madrid, 28040 Madrid, Spain; ‡ Instituto Madrileño de Estudios Avanzados en Nanociencia (IMDEA Nanoscience), C/Faraday, 9, 28049 Madrid, Spain

## Abstract

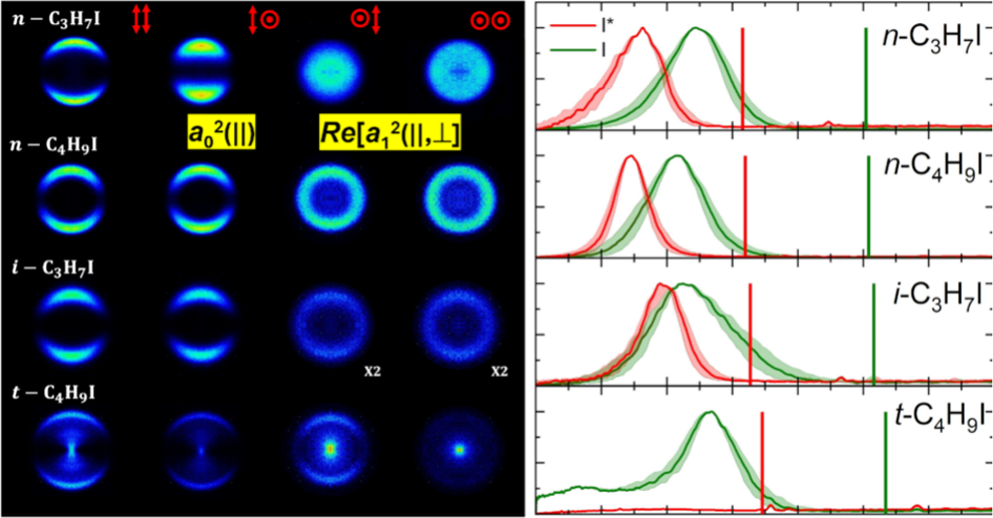

This work represents a step forward in the understanding of the widely studied photodynamics of alkyl iodides in the first absorption band. Ultraviolet (UV) photodissociation of several alkyl iodides (RI), specifically, a series of linear and ramified molecules with R = C_*n*_H_2*n*+1_, *n* = 1–4, at excitation wavelengths of 254 and 268 nm, which correspond to the maximum of the first absorption A-band, has been studied by combining resonance-enhanced multiphoton ionization (REMPI) detection of atomic photofragments I(^2^*P*_3/2_) and I*(^2^*P*_1/2_) and of pulsed slice imaging. Detailed examination of the total translational energy distributions of both atomic photofragments has been combined with stereodynamical information on the process obtained from the anisotropy β and alignment *a*_0_^2^(∥) and Re[*a*_1_^2^(∥, ⊥)] parameters to provide a description of the role played by the molecular structure of alkyl iodides in adiabatic and, especially, in nonadiabatic photodissociation dynamics through conical intersections or avoided crossings. The present results suggest that the linear structures couple more efficiently with the pure C–I reaction coordinate, whereas for the branched structures, the coupling with additional vibrational (bending) modes gains importance, showing the dissociation process a multidimensional character. In addition, a large degree of cofragment rotational alignment has been found for the small linear CH_3_I and C_2_H_5_I and, unexpectedly, for the branched *t*-C_4_H_9_I (*C*_3*v*_ symmetry), whereas the rest of the alkyl iodides show low alignment parameters.

## Introduction

Alkyl iodides have been considered over the years as model systems for photodissociation dynamics in UV.^1−10^ This family of molecules shows a first absorption band in the wavelength range of 220–320 nm with a maximum at 260 nm,^11^ which is termed the A-band. According to Mulliken,^1,2^ this A-band is assigned to a *n* → σ* transition from the I atom lone-pair orbital to an orbital centered on C–I of σ* antibonding character. The presence of the heavy I atom in the molecule implies strong spin–orbit (SO) coupling.^12^ There are three spin–orbit states that can be accessed by dipole-allowed transitions from the electronic ground state in *C*_3*v*_ symmetry. In the notation proposed by Mulliken,^1^ there are two states, ^3^*Q*_1_ and ^1^*Q*_1_, which are accessible through perpendicular transitions, and one state, ^3^*Q*_0_, which can be accessed by a parallel transition.^13^ Interestingly, the ^3^*Q*_0_ state correlates with the R + I*(^2^*P*_1/2_) fragments in an adiabatic way, while the former states correlate with R + I(^2^*P*_3/2_) fragments also adiabatically. In addition, there exists nonadiabatic coupling between the ^3^*Q*_0_ and ^1^*Q*_1_ states, which provides an additional means to produce I(^2^*P*_3/2_) products nonadiabatically.^4^ At the center of the absorption band, the parallel transition to the ^3^*Q*_0_ state dominates the absorption spectrum;^14^ at higher and lower wavelengths, respective perpendicular transitions to the ^3^*Q*_1_ and ^1^*Q*_1_ states gain importance.

**Figure 1:**
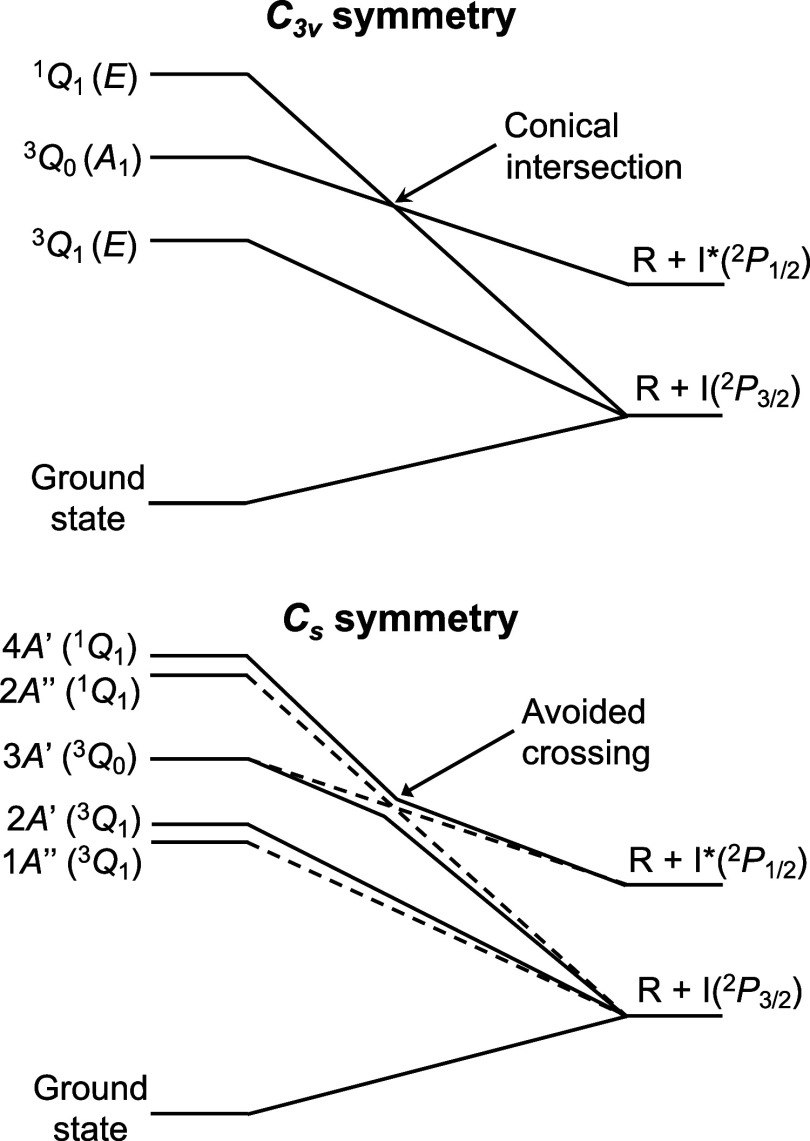
Diagrams showing the correlation of Mulliken’s ^3^*Q*_1_, ^3^*Q*_0_, and ^1^*Q*_1_ states of alkyl iodides along the reaction (C–I bond) coordinate for symmetries (top) *C*_3*v*_ and (bottom) *C*_*s*_.^15^

The proposed picture is strictly valid for symmetric-top alkyl iodides (*C*_3*v*_ symmetry), such as CH_3_I or *t*-C_4_H_9_I. For molecules of lower symmetry, as for instance *i*-C_3_H_7_I, the ^3^*Q*_1_ and ^1^*Q*_1_ states are split into two states of *A*′ and *A*″ symmetries, and the ^3^*Q*_0_ state changes symmetry to *A*′. This way, the ^3^*Q*_0_ state changes to the 3*A*′ state in *C*_*s*_ symmetry, while the ^1^*Q*_1_ and ^3^*Q*_1_ states split into 2*A*″ and 4*A*′ states, and 1*A*″ and 2*A*′ states, respectively. The correlation diagrams for alkyl iodides with *C*_3*v*_ and *C*_*s*_ symmetries^15^ are shown in Figure 1. In the case of *C*_*s*_ symmetry, the conical intersection that appears for *C*_3*v*_ symmetry is replaced by an avoided crossing between the 4*A*′ and 3*A*′ states. The former correlates adiabatically with the R + I*(^2^*P*_1/2_) fragments, while the latter is adiabatically correlated with the R + I(^2^*P*_3/2_) fragments. Importantly, nonadiabatic passage through this curve crossing retains a significant role in the photodissociation dynamics. Despite the symmetry loss, neither the calculated absorption spectra for the RI, nor the calculated ab initio potential energy curves show a significant dependence of the carbon-chain structure^16,17^ and thus, in all of the literature on the topic, the electronic states corresponding to the *C*_3*v*_ symmetry, i.e., ^3^*Q*_0_, ^3^*Q*_1_, and ^1^*Q*_1_, are used for convenience independently of the alkyl iodide under consideration.

In this work, the photodissociation of several saturated linear (CH_3_I, C_2_H_5_I, *n*-C_3_H_7_I, and *n*-C_4_H_9_I) and branched (*i*-C_3_H_7_I and *t*-C_4_H_9_I) alkyl iodides have been excited and dissociated at 254 and 268 nm close to the maximum of the first absorption *A*-band. The I*(^2^*P*_1/2_) and I(^2^*P*_3/2_) photoproducts are detected using the slice imaging technique in combination with (2 + 1) resonance-enhanced multiphoton ionization (REMPI) detection. The recorded iodine-atom images at different polarization directions of pump and probe lasers, and specifically the angular and translational energy distributions, are analyzed to provide information about the energy disposal into different degrees of freedom of the products as well as the dissociation atomic photofragment anisotropy. In the following sections, we will show how a direct comparison between sets of data taken at the two close excitation wavelengths challenges the traditional interpretation of the dissociation dynamics accepted for these molecular systems.

The maximum of the first *A* absorption band for different alkyl iodides found in the literature is as follows: CH_3_I at 258 nm,^18^ C_2_H_5_I at 258 nm,^19^*n*-C_3_H_7_I at 256 nm,^11^*n*-C_4_H_9_I at 257 nm,^20^*i*-C_3_H_7_I at 260 nm,^11^ and *t*-C_4_H_9_I at 268 nm.^21^ We have chosen the excitation wavelength 268 nm to compare the present results with those obtained from femtosecond time-resolved experiments by Corrales et al.^17^ and 254 nm to compare with femtosecond time-resolved experiments by Warne et al.^22^ and Downes-Ward et al.^23^ In particular, Warne et al.^22^ used time-resolved UV-pump (269 and 255 nm) and UV-probe (395 nm) multiphoton ionization photoelectron spectroscopy to study the photodissociation of CH_3_I close to the maximum of the A-band. They observed different reaction times associated with distinct dynamical structures at the two wavelengths studied. These surprising results were assigned to an unexpected contribution of the dynamics of the ^1^*Q*_1_ state at 255 nm, reflecting a more complex dissociation path occurring on the ^1^*Q*_1_ potential surface. Later, Downes-Ward et al.^23^ confirmed the same results at 254 nm by time-resolved measurements using an extreme ultraviolet probe in photoelectron spectroscopy experiments.

The paper is structured as follows. The experimental apparatus and a description of the analytical tools employed to extract the stereodynamical information are summarized in the Experimental Section. The results are presented and discussed in the corresponding sections.

## Experimental Section

The main specifications of the experimental apparatus and method have been widely described elsewhere.^24,25^ Here, only a brief description of the setup and experimental conditions will be given. All of the molecules used in this work have been obtained from Sigma-Aldrich with purities of 99%, except for *t*-C_4_H_9_I that was 95% pure. A molecular beam is created by expanding the vapor pressure of the studied molecule at room temperature using a pulse nozzle valve (General Valve Series 9, 0.5 mm orifice) with He (1 bar). The pulse gas traverses a 0.5 mm diameter skimmer (Beam Dynamics, Standard Model 2) and the resulting molecular beam enters into the ionization chamber, where it interacts with the photolysis and detection lasers. The two lasers are counter-propagated and focused with 25 cm focal lenses into a time-of-flight mass spectrometer (TOFMS). The pulse valve and lasers run with a repetition rate of 10 Hz.

To generate the photolysis laser radiation at the wavelengths used in this work, λ = 254 and 268 nm (located in the vicinity of the A-band maximum of the RI), a Nd:YAG (Quanta Ray Pro 230) pumped dye (Sirah Cobra-Stretch) laser is used, producing 2.2 mJ/pulse. With a delay time of 10 ns with respect of the photolysis pulse, the detection laser pulse (1.7 mJ/pulse, Nd:YAG (Quanta Ray Pro 190) pumped, frequency-doubled dye (Sirah Cobra-Stretch)) arrives at the interaction region. The two iodine atoms, I(^2^*P*_3/2_) and I*(^2^*P*_1/2_), are resonantly ionized through (2 + 1) REMPI schemes at 304.63 and 305.56 nm, respectively, which correspond to (^3^*P*_2_)6*p*[3]_5/2_ ← 5*p*(^2^*P*_3/2_) and (^3^*P*_1_)6*p*[1]_3/2_ ← 5*p*(^2^*P*_3/2_) transitions.

Slice images were acquired with a delayed-pulse extraction slicing setup using a single-field configuration.^24,26,27^ The delayed-pulse extraction permits the velocity spreading of the ion cloud in such a way that the arrival time of the cloud at the detector extends for hundreds of nanoseconds. Then, a narrow time gate is used in the detector, and thus, only the central slice of the expanded ion cloud was selected. The temporal width set for the gate must be as short as possible and in any case in the order of a few tens of nanoseconds, to ensure that the slice width corresponds only to the central portion of the sphere and any signal outside this central portion has been excluded. In the present case, the same temporal width was selected for all of the measurements. A 400 ns delay was applied on the repeller plate to measure I(^2^*P*_3/2_) and I*(^2^*P*_1/2_) images. The fragment ions were then accelerated to the detector by using a constant electric potential of 3.0 kV to the repeller plate.^24,27^ The ions then fly through a field-free time-of-flight region of 45 cm length before impacting impedance-matched microchannel plates (MCPs, Chevron configuration, 40 mm diameter). The resulting avalanche of electrons arrives at a phosphor screen (P47), generating a slice ion image. The image is acquired by a CCD camera (SONY 1024 × 768 pixels) driven by National Instrument (NI) LabView 7.1 and IMAQ VISION software. All raw slice images were symmetrized before extracting the kinetic energy and angular distributions from them. The resolution in velocity achieved was of about 1%.^26,27^

In order to study the stereodynamics of the photodissociation of different alkyl iodides studied, and due to the use of the slicing technique, the images for I(^2^*P*_3/2_), where atomic alignment effects are expected, have been recorded employing four linear pump–probe laser polarization configurations: *XX*, *XZ*, *ZX*, and *ZZ*. Here, *X* indicates a direction perpendicular to the laser propagation axis (which is *Y*), and *Z* stands for a direction parallel to the molecular beam axis. However, since the I*(^2^*P*_1/2_) fragment has total angular momentum *J* = 1/2 (and cannot show angular alignment), the corresponding I*(^2^*P*_1/2_) fragment images were acquired only for the case of pump and probe lasers parallel to the detector (i.e., *XX*).

For the calibration of the spectrometer, independent measurements were carried out using a single laser pulse experiment in which CH_3_I was excited, and CH_3_(*ν* = 0) fragments were detected by REMPI (2 + 1) at a wavelength of 333.45 nm, considering the well-known energetics for the CH_3_ + I(^2^*P*_3/2_) channel at this photolysis wavelength.

### Atomic Alignment Analysis

The slice imaging or slicing technique provides a useful tool to study the angular momentum alignment of photodissociation products.^28^ The slicing name refers to the possibility of detecting those photofragments localized in a narrow section—slice—of the photofragment 3D distribution. In particular, when the central slice is recorded, the consequent analysis does not require any mathematical transformation. The technique allows us to measure sliced images in different configurations of photolysis and detection laser polarization. The analysis of the photofragment polarization requires four pump–probe linear polarization configurations, namely, *XX*, *XZ*, *ZX*, and *ZZ*. Here, the *XYZ* axis system indicates the laboratory frame, with counter-propagating laser beams along the *Y*-axis. The molecular beam propagates along the *Z*-axis, which is the time-of-flight axis.

This way, the *XX* images contain the stereodynamic data: dissociation anisotropy and photoproduct alignment. In the case of *XZ* images, the polarization vector of the probe beam is perpendicular to the plane of the detector and the remaining β_2_^*XZ*^ parameter is associated with both the dissociation anisotropy and the photofragment polarization. In a similar way, the *ZX* images do not contain information related to the dissociation anisotropy. Finally, the *ZZ* images do not contain any dynamical information and are used as a reference to avoid systematic errors.

In the following paragraphs, the equations employed to perform a concerted analysis of the four images to provide different polarization parameters are presented.

The angular distributions extracted from the images are usually written as linear combinations of Legendre polynomials. If linearly polarized pump and probe laser pulses are used, then the linear combination is made of only even-order polynomials, and the polynomial expansion is limited by the number of pump and probe photons involved. For a one-photon dissociation process using linearly polarized light and a (2 + 1) REMPI detection, as used in the present work, a maximum rank of 6 results in a complete expression for the angular intensity^15^
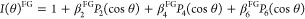
1where θ is the angle describing the photofragment distribution within the image plane. Here, *F* and *G* indicate the directions of the photolysis and probe lasers, respectively, with respect to *XY* (the slicing plane, which is perpendicular to time-of-flight axis *Z*). *P*_*k*_(cos θ) is the *k*th Legendre polynomial and β_k_^FG^ are the *k*th anisotropy parameters in the laboratory frame.

Equation 1 provides a convenient and directly measured set of β_*k*_^FG^ coefficients where all vector correlations are expressed in a standard way. Rakitzis and Zare^29,30^ proposed an ensemble of parameters, the *a*_*q*_^*k*^(*p*) molecular frame polarization parameters, and established general relationships with the laboratory frame anisotropy parameters, β_*k*_^FG^. For the particular case of pure perpendicular transitions, specific equations connecting β_*k*_^FG^ and *a*_*q*_^k^(⊥) were developed. A similar set of equations for parallel transitions have been developed recently by Recio et al.,^31^ involving the matching *a*_*q*_^*k*^(∥) parameters as well as the Re[*a*_*q*_^*k*^(∥, ⊥)] parameter, which accounts for the interference between parallel and perpendicular transitions. We will have *q* = 0 for a one-photon parallel transition (β = 2) and thus only *a*_0_^*k*^(∥) parameters. For angular distributions fitted to eq 1 with β_6_ = 0, *k* = 2, which results in the following expressions:
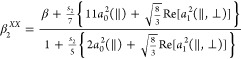
2a
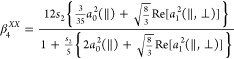
2b
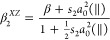
2c
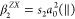
2d

It should be noted that for *k* = 2, the β_4_^*ZX*^ and β_4_^*XZ*^ parameters are identically zero. The *s*_2_ coefficient is a sensitivity factor, which can be obtained from Table 1 of ref (30). According to ref (32), the *s*_2_ coefficient shows a value of 80/333 for the I(^2^*P*_3/2_) photofragment when using a hyperfine depolarization coefficient of 10/37.

Limit values for the *a*_0_^2^(∥) parameter of the I(^2^*P*_3/2_) photofragment are in the range (−0.8, +0.8).^33^ Here, the higher values, positive and negative, correspond to the population in *m*_J_ = ±1/2 and *m*_J_ = ±3/2 states, with respect to the quantization of the I atom recoil direction.

In the case of a parallel transition, no net change in the projection of the total angular momentum on the bond axis is expected. For a diatomic molecule, the determination of the *a*_0_^2^(∥) parameter for one of the fragments provides similarly indirect information about the other, due to preservation of the angular momentum. However, if the coproduct of the atom is a polyatomic fragment, then the angular momenta of both photoproducts are conditioned by the rotation of the latter and no preferential population is expected for the atomic species. Any divergence from such an expectation would imply significant dynamical information.

## Results and Discussion

**Figure 2:**
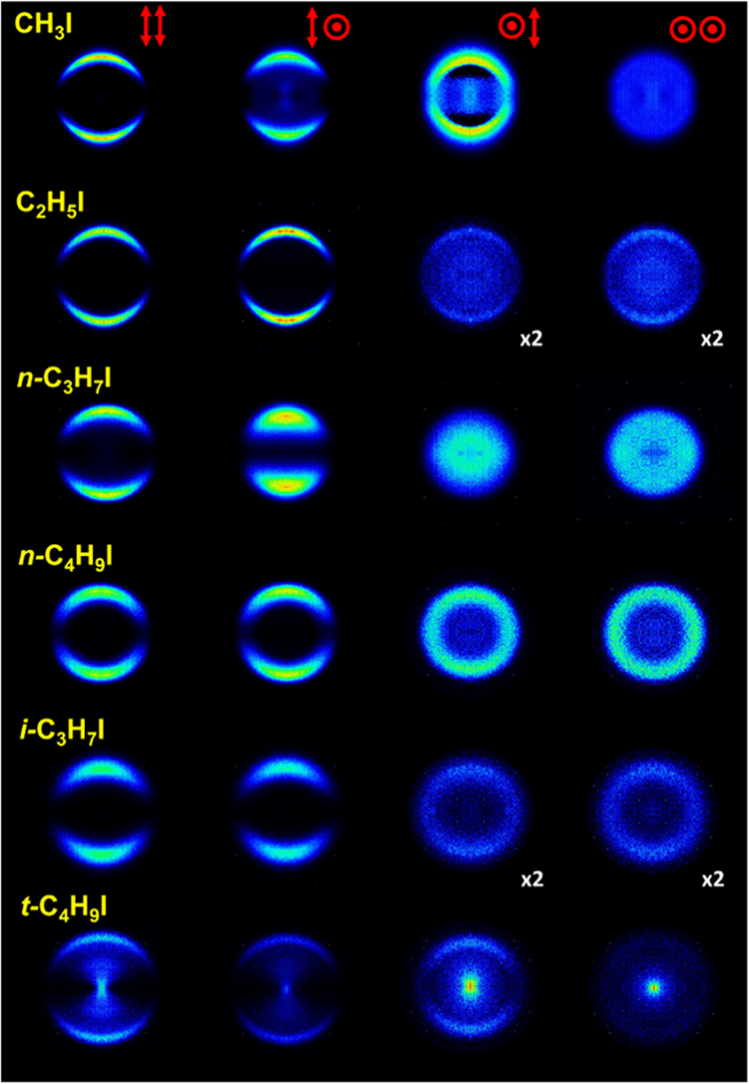
I(^2^*P*_3/2_) symmetrized slice images for the pump–probe polarizations *XX*, *XZ*, *ZX*, and *ZZ* (represented by vertical double arrows (*X* polarization) and circles *(Z* polarization)), measured at an excitation wavelength of 254 nm, and (2 + 1) REMPI probe wavelength 304.63 nm, for the six species studied in this work. The contrast in those figures marked with x2 has been enhanced by a factor of 2.

**Figure 3:**
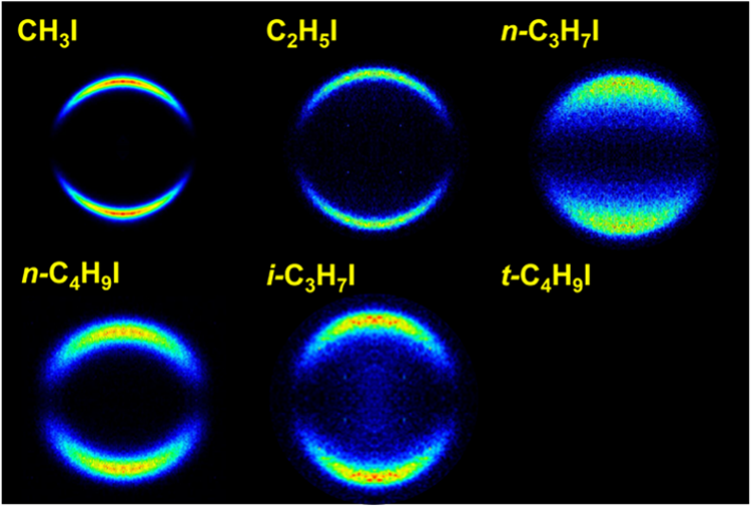
I*(^2^*P*_1/2_) symmetrized slice images (only for *XX* polarization configuration) measured at an excitation wavelength of 254 nm and (2 + 1) REMPI probe wavelength 305.56 nm, for five of the six species studied in this work. The *t*-C_4_H_9_I image is not shown since no signal was observed.

Figures 2 and 3 show a series of slice images recorded for the I(^2^*P*_3/2_) and I*(^2^*P*_1/2_) fragments, respectively, after dissociation of the corresponding alkyl iodides at the excitation wavelength of 254 nm, precisely at roughly the maximum of the A-band absorption spectra of the RI with the exception of branched *i*-C_3_H_7_I and *t*-C_4_H_9_I, for which the maximum is broader and shifted toward longer wavelengths, i.e., 260 and 268 nm, respectively.

Images of the I(^2^*P*_3/2_) fragment at the four polarization configurations were recorded for each species, CH_3_I, C_2_H_5_I, *n*-C_3_H_7_I, *n*-C_4_H_9_I, *i*-C_3_H_7_I, and *t*-C_4_H_9_I. The polarization configurations are indicated by red arrows (*X* polarization) and circles (*Z* polarization) at the top of Figure 2. The *XX* images, which gather the information on the dissociation event and all possible polarization effects, show (with the exception of *t*-C_4_H_9_I) a single feature, i.e., a highly anisotropic ring that can be ascribed to the formation of I fragments by absorption through a parallel transition and further R–I bond excision in a highly repulsive potential energy surface.^17^ The width of the ring increases as the carbon chain gains complexity, with the exception, once more, of *t*-C_4_H_9_I, which surprisingly shows a substantially narrower ring than its linear equivalent (*n*-C_4_H_9_I). The I(^2^*P*_3/2_) image arising from photodissociation of *t*-C_4_H_9_I shows, additionally, a feature at lower recoil energies (inside the ring), which can be associated with multiphoton ionization and subsequent dissociation.^17^

Comparison between the images taken at the four polarizations would shed some light on the I(^2^*P*_3/2_) photofragment alignment. Differences between the *XX*, *XZ*, and *ZX* images are attributable to photofragment polarization effects. In particular, similar intensity distributions of *XX* and *XZ* images might suggest that the stereodynamics is governed by the dissociation process and that the polarization effects, if present, would contribute mildly. Such could be the case for C_2_H_5_I, *n*-C_4_H_9_I, *i*-C_3_H_7_I, and *t*-C_4_H_9_I species. The CH_3_I and *n*-C_3_H_7_I species show, however, large variations in the intensity distributions of the *XX*, *XZ*, and *ZX* images, which suggests a significant photofragment alignment for those molecules.

Since no alignment can be observed for the I*(^2^*P*_1/2_) fragment, Figure 3 shows only *XX* images for each molecule. In this case, all of the recorded images can be described by a single feature, a highly anisotropic ring corresponding to a parallel transition, that has been assigned to prompt dissociation in a highly repulsive surface.^17^ Clearly, the width of the ring depends strongly on the structure of the molecule, although in general terms it can be linked to the number of carbon atoms in the chain and the complexity of the species. No signal attributed to the I*(^2^*P*_1/2_) fragment was detected in the photodissociation of *t*-C_4_H_9_I.

### Total Translational Energy Distributions

**Figure 4:**
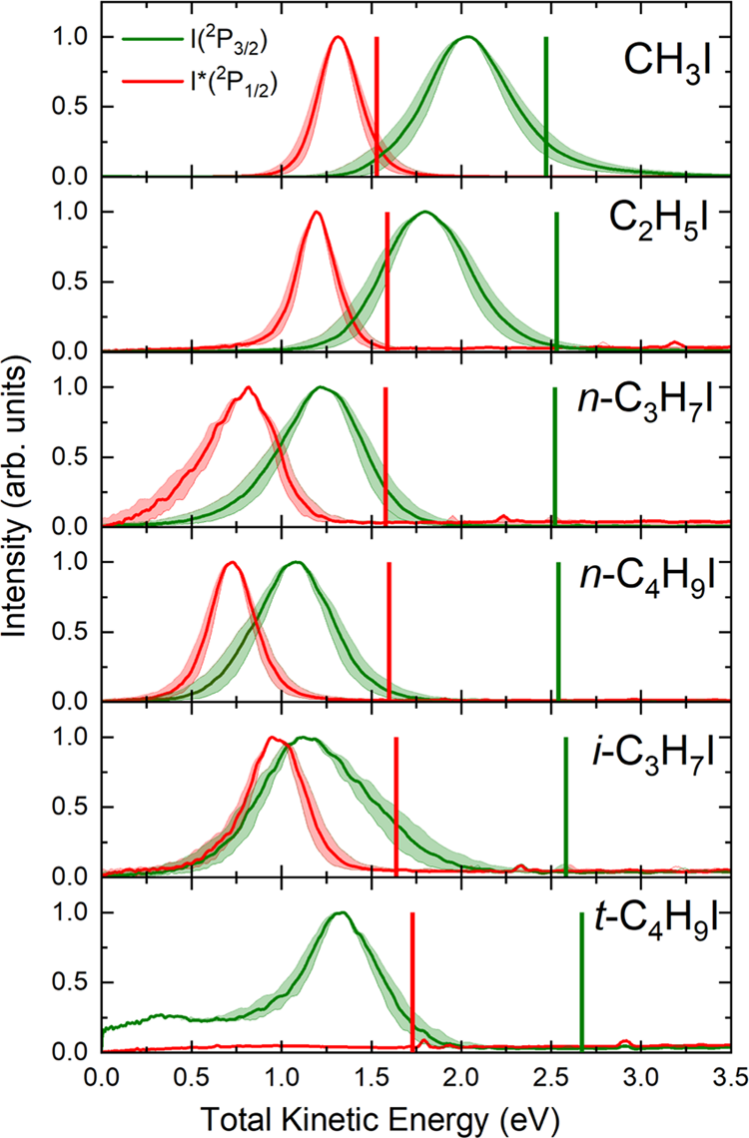
Comparison between I(^2^*P*_3/2_) (green) and I*(^2^*P*_1/2_) (red) total translational energy distributions (TEDs) after the photodissociation of different alkyl iodides at an excitation wavelength of 254 nm. The vertical green and red lines indicate the maximum total energy available for both dissociation channels yielding I(^2^*P*_3/2_) and I*(^2^*P*_1/2_) in correlation with *R*, respectively. The shadowed areas indicate the energy profile of the slice of the sphere measured.

Angular integration of *XX* images shown in Figures 2 and 3 for both fragments I(^2^*P*_3/2_) and I*(^2^*P*_1/2_) renders the total translational energy distributions (TEDs) shown in Figure 4. The reference (vertical line) represents the total available energy for the dissociation products after C–I bond cleavage for each channel for different alkyl iodides RI, which has been calculated as follows
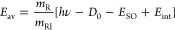
3where *m*_R_ and *m*_RI_ are the masses of the R cofragment and the parent molecule RI, respectively, *h*ν represents the photon energy, i.e., the excitation energy transferred to the molecule, *E*_SO_ corresponds to the I(^2^*P*) spin–orbit splitting (i.e., 0 and 0.943 eV for I(^2^*P*_3/2_) and I*(^2^*P*_1/2_), respectively), and *E*_int_ is the internal energy of the RI, to be considered negligible in a supersonic expansion. The term *D*_0_ denotes the C–I bond dissociation energy for each RI. The *D*_0_ values have been reported in the literature and are collected in Table 1. With the exception of *t*-C_4_H_9_I, the distributions display a simple Gaussian-like shape, suggesting a single dissociation mechanism in every case. The I*(^2^*P*_1/2_) distribution is absent in the panel corresponding to *t*-C_4_H_9_I photodissociation, and the I(^2^*P*_3/2_) distribution shows, in addition, a broad feature at low total translational energies, corresponding to the abovementioned MPI process.^17^

**Table 1:** Experimental Dissociation Energy (*D*_0_) Taken from Refs (34) and (37) (in eV), Fraction of the Available Energy That Appears as R Cofragment Internal Energy (*f*_int_), and Area Under the Curve of the Total Translational Energy Distributions (TEDs) (in Arbitrary Units) for Both Photodissociation Channels Yielding I(^2^*P*_3/2_) and I*(^2^*P*_1/2_) at Excitation Wavelengths of 254 and 268 nm for the Alkyl Iodides Studied in This Work^a^

			I*(^2^*P*_1/2_)				I(^2^*P*_3/2_)		
		254 nm		268 nm		254 nm		268 nm	
	*D*_0_	*f*_int_	area	*f*_int_	area	*f*_int_	area	*f*_int_	area
CH_3_I	2.41^34^	0.13	0.29	0.08	0.24	0.17	0.59	0.03	0.45
C_2_H_5_I	2.35^36^	0.25	0.28	0.19	0.47	0.29	0.59	0.30	0.80
*n*-C_3_H_7_I	2.36^37^	0.48	0.47	0.37	0.28	0.52	0.56	0.47	0.55
*n*-C_4_H_9_I	2.34^35^	0.54	0.32	0.54	0.32	0.58	0.52	0.58	0.52
*i*-C_3_H_7_I	2.30^37^	0.42	0.42	0.61	0.11	0.56	0.72	0.59	0.77
*t*-C_4_H_9_I	2.21^35^					0.43	0.41	0.46	0.43

a The experimental error is estimated to be about 15% for each parameter.

A striking feature in Figure 4 is the shift of the distributions toward lower-energy values as the linear carbon chain increases in size, while the opposite trend is observed for the branched species, *i*-C_3_H_7_I and *t*-C_4_H_9_I. The displacement of the distribution from the available energy vertical mark (taking the peak of the distribution as a reference) is associated with the amount of available energy transferred to internal degrees of freedom (rotation and vibration) of R cofragments. A peak of the distribution that is close to the maximum available energy indicates a photodissociation where most of the energy available for the C–I bond cleavage is transformed into fragment kinetic energy. In contrast, a distribution shifted downward from the available energy reference indicates that a certain amount of the available energy has been transferred to rotational and vibrational (internal) energy of the cofragments, *E*_int_^′^. The described energy distribution can be quantified by the fraction of the available energy that appears as cofragment internal energy, *f*_int_, which can be obtained as
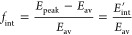
4

The *f*_int_ values determined from the TEDs for different RIs are collected in Table 1 along with the areas of the Gaussian-like curves describing the total TEDs. The area of the TEDs correlates straightforwardly with the internal energy of the R cofragment if, in addition, its molecular structure is considered. As the number of carbon atoms in the R chain increases or if the structure changes from linear to branched, the number of vibrational degrees of freedom (normal modes) with a variety of frequencies increases as well. Larger areas would indicate R radicals of increasing complexity, recoiling with higher rotational and/or vibrational excitation in an increasing number of vibrational normal modes. On the other hand, lower areas would indicate R radicals with a simpler structure, with fewer vibrational degrees of freedom, and which barely rotate or vibrate when recoiling. Intramolecular vibrational energy redistribution (IVR) should have an increasing role as the number of vibrational normal modes increase in the R cofragment, especially in the nonadiabatic photodissociation dynamics mediated by the conical intersection (*C*_3*v*_ symmetry) or the avoided crossing (*C*_*s*_ symmetry).

The trends observed for the *f*_int_ values and the areas of the curves in the TEDs measured at 254 nm for both dissociation channels and for different linear and branched RI clearly indicate the distinct internal energy content of the R cofragment resulting from the photodissociation depending on the molecular structure and the effect of molecular complexity on the nonadiabatic dynamics characteristic of the photodissociation mechanism associated with alkyl iodides.

In particular, at 254 nm excitation energy and for the R + I(^2^*P*_3/2_) channel, *f*_int_ values increase almost linearly for the linear RI, from zero for CH_3_I up to 0.58 for *n*-C_4_H_9_I. Conversely, the areas of the TED curves slightly decrease along the series of linear RI. For the R + I*(^2^*P*_1/2_) channel, *f*_int_ values increase also linearly for the linear RI, from zero for CH_3_I up to 0.54 for *n*-C_4_H_9_I, and the areas of the TED curves barely increase or level off. The trend is quite different for the R + I(^2^*P*_3/2_) channel of the branched RI (*i*-C_3_H_7_I and *t*-C_4_H_9_I), i.e., the *f*_int_ values decrease and the areas of the TEDs also decrease strongly.

The measurements described above were also carried out at an excitation wavelength of 268 nm, which is shifted to red with respect to the maximum of the *A*-band absorption spectra of the RI with the exception of *t*-C_4_H_9_I for which the maximum of the absorption band is precisely at 268 nm.^21^

**Figure 5:**
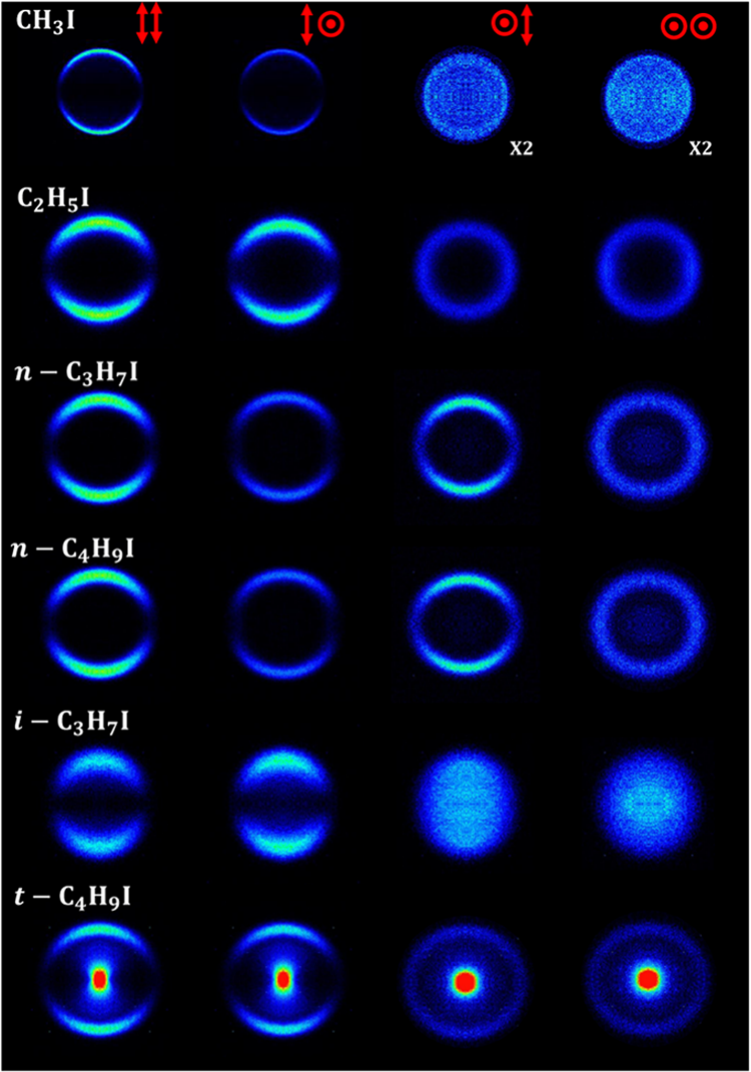
I(^2^*P*_3/2_) symmetrized slice images for the pump–probe polarizations *XX*, *XZ*, *ZX*, and *ZZ* (represented by vertical double arrows (*X* polarization) and circles (*Z* polarization)) measured at an excitation wavelength of 268 nm and (2 + 1) REMPI probe wavelength of 304.63 nm, for the six species studied in this work. The contrast in those figures marked with x2 has been enhanced by a factor of 2.

**Figure 6:**
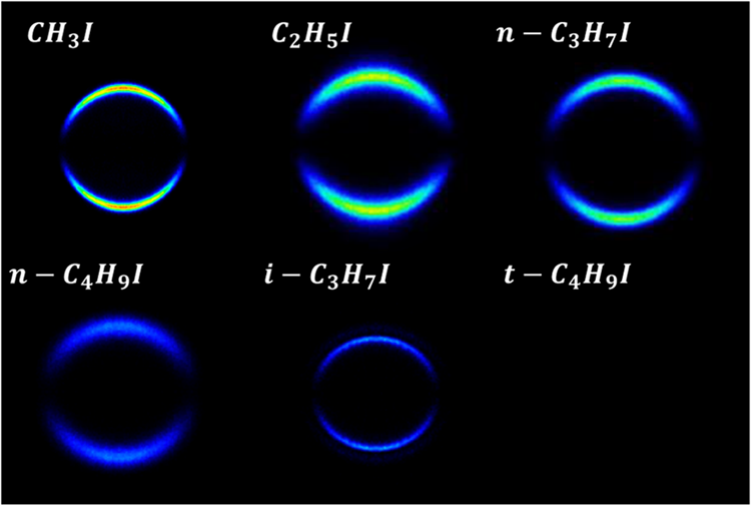
I*(^2^*P*_1/2_) symmetrized slice images (only for *XX* polarization configuration) measured at an excitation wavelength of 268 nm and (2 + 1) REMPI probe wavelength of 305.56 nm for five of the six species studied in this work. The *t*-C_4_H_9_I image is not shown since no signal was observed.

**Figure 7:**
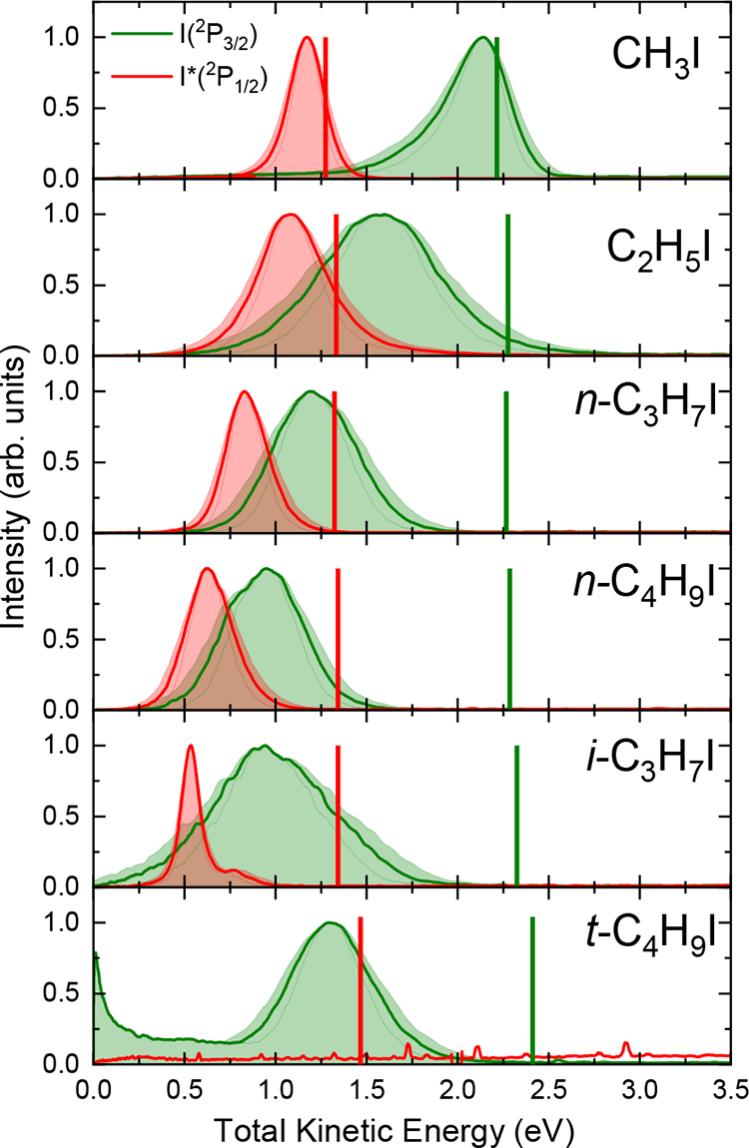
Comparison between the I(^2^*P*_3/2_) (green) and I*(^2^*P*_1/2_) (red) total translational energy distributions (TEDs) after the photodissociation of different alkyl iodides at an excitation wavelength of 268 nm. The vertical green and red lines indicate the maximum total energy available for both dissociation channels yielding I(^2^*P*_3/2_) and I*(^2^*P*_1/2_) in correlation with R, respectively. The shadowed areas indicate the energy profile of the slice of the sphere measured.

The images recorded for the I(^2^*P*_3/2_) fragment at the four polarization configurations for different RI studied upon excitation at 268 nm are shown in Figure 5, while the I*(^2^*P*_1/2_) images are shown (only for the *XX* polarization configuration) in Figure 6. The corresponding total TEDs for both fragments are displayed in Figure 7. Taking into consideration that the two excitation wavelengths employed in this study are close to each other and to the absorption maximum of the A-band of different RI, similar images would be rather expected. Certainly, for both excitation wavelengths, an anisotropic ring, whose width and radius depend strongly on the molecular structure and photodissociation channel (adiabatic or nonadiabatic), constitutes the single feature of most of the slice images. The differences in the width and size of the rings can be clearly detected in the total TEDs of Figures 4 and 7. Interestingly, the most striking difference is found actually in the ring anisotropy. A simple visual inspection of Figures 2 and 5, on the one hand, and of Figures 3 and 6, on the other hand, indicates that the anisotropy of the I(^2^*P*_3/2_) rings is lower at 268 nm than at 254 nm, while the opposite occurs for the I*(^2^*P*_1/2_) fragment. Specific values of the dissociation anisotropy parameter will be presented and discussed in the next section.

At 268 nm, the *f*_int_ values shown in Table 1, reflecting the shift of the total TED maximum with respect to the available energy reference, increase for both dissociation channels (I*(^2^*P*_1/2_) and I(^2^*P*_3/2_)) when the complexity of the linear RI increases, in a similar fashion as at 254 nm. Therefore, for both 254 and 268 nm excitation wavelengths, the internal energy of the R cofragment increases as the chain increases in the series of linear RI. As commented on above, more energy is released as internal energy and less as translational energy of the fragments as the vibrational degrees of freedom and size of the linear R cofragment increase. Moreover, this effect is stronger for the nonadiabatic R + I(^2^*P*_3/2_) channel, and this explains why for the largest linear molecule *n*-C_4_H_9_I, the two channels overlap for both 254 and 268 nm excitation wavelengths. This result also explains why the rings in the images become broader as the size and complexity of the R cofragment increase.

As in the case of a 254 nm excitation wavelength, the branched molecules show a different trend in comparison with the linear ones. Now, *f*_int_ values stabilize (*i*-C_3_H_7_I) or even decrease (*t*-C_4_H_9_I).^17^ Thus, the amount of total energy released as fragment translational energy increases for the branched molecules, which may correspond to a less efficient energy flux into the internal degrees of freedom of the branched radicals. These results perfectly match those obtained previously in femtosecond time-resolved experiments at 268 nm.^17^

Similar trends for the areas of the curves in the TEDs are observed for both dissociation channels at 268 nm in comparison with the 254 nm excitation wavelength.

The alkyl iodides studied in this work might be vibrationally characterized by four main vibrational modes. The stretching mode of the C–I bond is primarily excited in the absorption step, leading to fast C–I dissociation. The transition to the ^3^*Q*_0_/3*A*′ state implies, however, a geometrical change that would reflect in the bending C–I mode.^21^ The internal energy accumulated in these two main vibrational modes is, in addition, partially transferred to the carbonated chain through intramolecular vibrational energy redistribution (IVR). The vibrational activity in the carbonated chain might be visualized in terms of two major modes (or group of modes), which we will call, for conciseness, the C–C stretching mode, responsible for the propagation of the vibrational excitation through the R moiety, and the C–C bending mode, which would include the C–H motion as well. The amount of internal energy transferred to the dissociation products will strongly depend on the interplay of the four vibrational modes, which, in turn, is plainly related to the length and structure of the molecule under scrutiny.

As the molecule gets heavier and branched, the amount of internal energy transferred from the stretching C–I to the bending C–I increases, a fact that surfaces as an increasing shift in the corresponding translational energy distributions. One intriguing aspect of the photodissociation dynamics is that the energy transferred from the C–I coordinate to the C–C stretching and bending modes is not just accumulated as vibrational activity of different internal modes, but it does contribute as well to the dissociation process, providing to the dissociation a multidimensional character.^21^

The observed trend for *f*_int_ and for the areas of the curves in the TEDs at both wavelengths informs us about the coupling between the C–I modes and the C–C and C–H internal modes. For the linear molecules, the increase of *f*_int_ along the series of linear molecules indicates an efficient transfer of energy along the C–C chain and the pertinent redistribution among the low-energy vibrational modes, which will result, as will be discussed later, in a progressive scrambling of the R-fragment along the recoil axis. Remarkably, there are no significant differences between the adiabatic and nonadiabatic channels, which indicates that the IVR takes place primarily prior to the adiabatic passing through the conical intersection. For the branched molecules, the *f*_int_ measured for *i*-C_3_H_7_I is similar to that of its linear counterpart and larger than that of *t*-C_4_H_9_I. These values suggest that the redistribution of the internal energy along the carbonated chain affects mostly the skeleton C–C bending modes (and probably, in the case of *i*-C_3_H_7_I, the lower-energy C–H vibrational modes), which are straightforwardly excited through the C–I stretching motion.

### Dissociation Anisotropy and Alignment Parameters

The angular distributions of the I(^2^*P*_3/2_)/I*(^2^*P*_1/2_) photofragments obtained from the radial integration of the slice images depicted in Figures 2, 3, 5, and 6 have been fitted to eq 1, which is valid for a one-photon dissociation process and (2 + 1) REMPI photofragment detection.

The least-squares fit generates a set of anisotropy parameters (β_2_, β_4_, and β_6_) for each image. In the case of the I*(^2^*P*_1/2_) fragment, the total angular momentum is *J* = 1/2, and no alignment is present. Thus, only the *XX* images were acquired, which were consequently fitted using a single β_2_ parameter in eq 1 (i.e., the sum is truncated to the second term). However, for total angular momentum *J* = 3/2, as for the I(^2^*P*_3/2_) fragment, *J* can be produced preferentially in the *m*_*J*_ = ±1/2 and ±3/2 states. Accordingly, the angular distributions extracted from the *XX* images were fitted to the β_*i*_ terms of eq 1. Only one β_2_ parameter was needed for the *XZ* and *ZX* images, whereas the β_6_ parameter was found to be close to zero in all cases. The β_2_ and β_4_ anisotropy parameters obtained from the fits of the *XX*, *XZ*, and *ZX* slice images for the I(^2^*P*_3/2_) fragment at the two excitation wavelengths and for the series of alkyl iodides studied are listed in Table 2.

**Table 2:** β_2_ and β_4_ Anisotropy Parameters Obtained from the Fits of the *XX*, *XZ*, and *ZX* Slice Images for I(^2^*P*_3/2_) Measured at Excitation Wavelengths of 254 and 268 nm for the Series of Alkyl Iodides Studied in This Work^a^

	254 nm				268 nm			
	β_2_^*XX*^	β_4_^*XX*^	β_2_^*XZ*^	β_2_^*ZX*^	β_2_^*XX*^	β_4_^*XX*^	β_2_^*XZ*^	β_2_^*ZX*^
CH_3_I	2.34	0.41	1.93	0.56	1.75	–0.19	1.65	–0.05
C_2_H_5_I	1.89	–0.23	1.78	0.00	1.71	–0.17	1.57	–0.29
*n*-C_3_H_7_I	1.95	–0.13	2.31	0.21	1.82	–0.07	1.57	0.75
*n*-C_4_H_9_I	1.83	–0.01	1.85	0.17	1.79	–0.08	1.82	0.08
*i*-C_3_H_7_I	2.04	0.09	2.03	0.06	1.76	–0.04	1.78	0.58
*t*-C_4_H_9_I	2.00	0.10	1.86	0.44	2.05	0.40	2.00	0.50

a The uncertainties for the βi parameters are estimated to be within ±0.2 for CH_3_I and ±0.05 for all of the other species.

Table 3 shows the dissociation anisotropy parameter β for the I*(^2^*P*_1/2_) fragment, and β and the polarization parameters *a*_0_^2^(∥) and Re[*a*_1_^2^(∥, ⊥)], extracted from the phenomenological β_*i*_ parameters of Table 2 using eq 2, for the I(^2^*P*_3/2_) fragment. At first glance, the values obtained for β agree, in all cases, with a fast prompt photodissociation mechanism occurring from the ^3^*Q*_0_ or 3*A*′ states for *C*_3*v*_ or *C*_*s*_ symmetries, respectively, for both R + I(^2^*P*_3/2_) and R + I*(^2^*P*_1/2_) channels.^16,34^ The large positive values, close to the maximum β = 2 corresponding to a pure parallel transition, prove that at the two excitation wavelengths employed in this work and for all of the alkyl iodides studied, the absorption step is produced almost exclusively to the ^3^*Q*_0_/3*A*′ state.

**Table 3:** Anisotropy Parameter (β) for Both Iodine Fragments, I*(^2^*P*_1/2_) and I(^2^*P*_3/2_), and Alignment Parameters (*a*_0_^2^(∥), Re[*a*_1_^2^(∥, ⊥)]) Derived from the I(^2^*P*_3/2_) Images for Each of the Studied Alkyl Iodides Measured at Excitation Wavelengths of 254 and 268 nm^a^

	I*(^2^*P*_1/2_)		I(^2^*P*_3/2_)					
	254 nm	268 nm	254 nm			268 nm		
	β	β	β	*a*_0_^2^(∥)	Re[*a*_1_^2^(∥, ⊥)]	β	*a*_0_^2^(∥)	Re[*a*_1_^2^(∥, ⊥)]
CH_3_I	2.38	1.97	2.17	1.07	–0.04	1.56	0.45	0.08
C_2_H_5_I	1.64	2.18	1.79	0.53	0.08	1.60	0.15	0.02
*n*-C_3_H_7_I	1.87	2.00	1.90	0.05	–0.01	1.64	0.27	0.02
*n*-C_4_H_9_I	1.68	1.75	1.85	–0.09	0.00	1.80	–0.04	0.00
*i*-C_3_H_7_I	2.12	1.90	2.03	0.05	–0.02	1.71	0.13	0.02
*t*-C_4_H_9_I			1.87	0.70	0.01	2.00	0.53	–0.06

a The uncertainties for the β and *a*_0_^2^(∥) and Re[*a*_1_^2^(∥, ⊥)] parameters are estimated to be within ±0.2 for CH_3_I and ±0.05 for all other species.

It must be noticed, however, that the anisotropy parameter associated with iodine images measured from CH_3_I photodissociation is over the limiting value for a parallel transition. The iodine images that correspond to the photodissociation of CH_3_I at 254 nm excitation wavelength, shown in Figures 2 and 3, are affected by an unavoidable nonnegligible space charge. This space charge effect has been considered in the data analysis, but, in any case, it entails an overestimation of the anisotropy parameters for a parallel transition.

The differences between the various RIs have been traditionally attributed to the length and structure of the radical R moiety. In the present case, general trends are observed for the β parameter as a function of the linear and branched RI molecules irrespective of the excitation wavelength. In particular, a shallow decrease of β from the limiting value of +2 toward values between +1.5 and +1.8—reflecting the interplay of the conical intersection (or avoided crossing) that connects the ^3^*Q*_0_-like state with the R + I(^2^*P*_3/2_) channel—occurs as the length of the carbon chain for the linear RI increases, irrespective of the dissociation channel and excitation wavelength. Interestingly, for the branched RI, the β values tend to increase (with respect to their linear counterpart), reaching values close to or equal to the limiting value of +2.

The *a*_0_^2^(∥) alignment parameters for the I(^2^*P*_3/2_) photofragment shown in Table 3 vary from +0.56, close to the limiting value of +0.8, for CH_3_I, down to around zero for the longer linear RI, *n*-C_3_H_7_I and *n*-C_4_H_9_I, at the excitation wavelength of 254 nm. At 268 nm, *a*_0_^2^(∥) also decreases for the same sequence of linear RIs. Interestingly, the *a*_0_^2^(∥) values for the branched *i*-C_3_H_7_I are comparable to those of its linear counterpart *n*-C_3_H_7_I. For the branched *t*-C_4_H_9_I, the *a*_0_^2^(∥) value at 254 nm is close to the limit (+0.7), and it is significantly large (+0.53) at 268 nm. At both excitation wavelengths, the large *a*_0_^2^(∥) values, which are in the same trend as for CH_3_I, would indicate that a high alignment is recovered when there is a higher symmetry in the molecule (*C*_3*v*_ vs *C*_*s*_) irrespective of the length and structure of the R radical.

A reduction of *a*_0_^2^(∥) with respect to asymptotic values (±0.8) in a polyatomic system would imply an increase of the population of internal (vibrational) states in the photodissociation process by an increase of IVR of the available energy. The increase of IVR would entail a quality loss of the C–I bond as a quantization axis. In other words, low *a*_0_^2^(∥) values would correspond to scrambling of the R moiety along the recoil direction.

As seen in Table 3, the *a*_0_^2^(∥) parameter decreases along the series of linear RI molecules. For CH_3_I and C_2_H_5_I, the *a*_0_^2^(∥) parameter takes large positive values at both excitation wavelengths, indicating a preference for the *m*_J_ = ± 3/2 states of the I(^2^*P*_3/2_) fragment. Taking into account the limiting values of ±0.8 for this parameter, the *a*_0_^2^(∥) obtained for CH_3_I and C_2_H_5_I imply a high degree of alignment in the corresponding coproducts in each case. Such a result turns out to be exceptional if we consider the polyatomic nature of both CH_3_ and C_2_H_5_ fragments. The alignment of the I(^2^*P*_3/2_) atom implies that the projection of the CH_3_ and C_2_H_5_ rotational angular momentum is greatly constrained along the recoil direction. This constraint implies that those fragments do not isotropically mix, and the rotational angular momentum shows instead a narrow distribution of rotational states, which are aligned along the recoil direction. As the linear chain increases, the alignment decreases and it is totally lost for *n*-C_4_H_9_I at both excitation wavelengths. The dependence of *a*_0_^2^(∥) with the length of the carbonated chain is in excellent agreement with the values measured for *f*_int_ discussed in the preceding section and suggests that the C–I coordinate is mainly coupled with the normal skeleton C–C vibrational modes.

Strikingly, a large *a*_0_^2^(∥) value is obtained for *t*-C_4_H_9_I at both excitation wavelengths, which indicates a large degree of alignment along the recoil direction. With similar *f*_int_ values than its linear counterpart, the presence of an extra methyl moiety in the central carbon atom of the *t*-C_4_H_9_ fragment prevents efficient excitation of C–C bending modes and, therefore, the vibrational excitation initially located in the C–I bond is transferred toward the C–H modes with little deformation of the chain.

The Re[*a*_1_^2^(∥, ⊥)] parameter has been measured as well and the corresponding values are gathered in Table 3. In all cases, the Re[*a*_1_^2^(∥, ⊥)] values are close to zero, although those numbers might not reflect necessarily valuable information. The Re[*a*_1_^2^(∥, ⊥)] parameter provides information about coherent contributions from the interference between parallel and perpendicular transitions. Such interference might indeed exist and be present in the molecules studied in this work yet not show up at the studied excitation wavelengths. As reported by Rakitzis et al.,^38^ while *a*_0_^*k*^ is practically independent of the excitation wavelength, the Re[*a*_1_^2^(∥, ⊥)] interference parameter displays an oscillatory behavior with the excitation wavelength. Without more data at different excitation wavelengths to validate the present results, we cannot extract any valid conclusion from the Re[*a*_1_^2^(∥, ⊥)] values shown in Table 3.

## Conclusions

The photodissociation of linear and branched alkyl iodides in the *A*-band has been studied by slice imaging and resonance-enhanced multiphoton ionization of the iodine fragments at the two excitation wavelengths of 254 and 268 nm in the vicinity of the maximum of the absorption band. The results presented in this work highlight the effect of the carbonated chain (R) length and structure on the photofragment energy distribution and anisotropy. It has been shown how the vibrational modes of the carbon chain distribute efficiently the energy deposited in the C–I bond after absorption of the excitation photon by the molecules. For the linear species, the increase of the fraction of the available energy that is transferred to internal degrees of freedom of the molecule, *f*_int_, increases along the series, while the R cofragment rotational alignment, represented by the *a*_0_^2^(∥) parameter, decreases accordingly. Remarkably, the smaller CH_3_I and C_2_H_5_I species show a large degree of cofragment rotational alignment along the recoil direction. This alignment is lost for the longer molecules as the content of the fragment’s internal energy increases. The *f*_int_ values for the branched species, on the other hand, show a mild decrease with the size of the molecule and a certain stabilization with respect to their linear counterparts, indicating that the energy redistribution is not favored when the C–I stretch mode is normal to the carbon chain. In this sense, the case of *t*-C_4_H_9_I at 254 nm is particularly interesting, showing the largest alignment of the ensemble.

## References

[ref1] Mulliken R. S. Intensities in Molecular Electronic Spectra X. Calculations on Mixed-Halogen, Hydrogen Halide, Alkyl Halide, and Hydroxyl Spectra. J. Chem. Phys. 1940, 8, 382 10.1063/1.1750671.

[ref2] Mulliken R. S.; Teller E. Interpretation of the methyl iodide absorption bands near λ 2000. Phys. Rev. 1942, 61, 283 10.1103/PhysRev.61.283.

[ref3] Donohue T.; Wiesenfeld J. R. Photodissociation of alkyl iodides. J. Chem. Phys. 1975, 63, 3130 10.1063/1.431741.

[ref4] Riley S. J.; Wilson K. R. Excited fragments from excited molecules: energy partitioning in the photodissociation of alkyl iodides. Faraday Discuss. Chem. Soc. 1972, 53, 132 10.1039/dc9725300132.

[ref5] Guo H.; Schatz G. C. Time-dependent dynamics of methyl iodide photodissociation in the first continuum. J. Chem. Phys. 1990, 93, 393 10.1063/1.459538.

[ref6] Guo H.; Lao K. Q.; Schatz G. C.; Hammerich A. D. Quantum nonadiabatic effects in the photodissociation of vibrationally excited CH_3_I. J. Chem. Phys. 1991, 94, 6562 10.1063/1.460283.

[ref7] Rist C.; Alexander M. H. Adiabatic representations for the study of flux redistribution during photodissociation involving coupled electronic states: The effect of vibrational excitation on the photofragmentation of CH_3_I. J. Chem. Phys. 1993, 98, 6196 10.1063/1.464813.

[ref8] Schinke R. Photodissociation Dynamics; Cambridge University Press: N.Y., 1993.

[ref9] Amatatsu Y.; Morokuma K.; Yabushita S. Ab initio potential energy surfaces and trajectory studies of A-band photodissociation dynamics: CH_3_I* → CH_3_ + I and CH_3_ + I*. J. Chem. Phys. 1991, 94, 4858 10.1063/1.460571.

[ref10] Amatatsu Y.; Yabushita S.; Morokuma K. Full nine-dimensional ab initio potential energy surfaces and trajectory studies of A-band photodissociation dynamics: CH_3_I* → CH_3_ + I, CH_3_ + I*, and CD_3_I* → CD_3_ + I, CD_3_ + I*. J. Chem. Phys. 1996, 104, 9783 10.1063/1.471758.

[ref11] Roehl C. M.; Burkholder J. B.; Moortgat G. K.; Ravishankara A. R.; Crutzen P. J. Temperature dependence of UV absorption cross sections and atmospheric implications of several alkyl iodides. J. Geophys. Res.: Atmos. 1997, 102, 12819 10.1029/97JD00530.

[ref12] Herzberg G. Molecular Spectra and Molecular Structure; Van Nostrand Company, 1996.

[ref13] Mulliken R. S. The Low Electronic States of Simple Heteropolar Diatomic Molecules. I. General Survey. Phys. Rev. 1936, 50, 1017 10.1103/PhysRev.50.1017.

[ref14] Riley S. J.; Wilson K. R. Magnetic circular dichroism spectra of the methyl halides. Resolution of the n→ σ* continuum. Chem. Phys. Lett. 1975, 34, 39.

[ref15] McGivern W. S.; Li R.; Zou P.; North S. W. Photodissociation dynamics of CH_2_BrCl studied using resonance enhanced multiphoton ionization (REMPI) with time-of-flight mass spectrometry. J. Chem. Phys. 1999, 111, 5771 10.1063/1.479874.

[ref16] Shubert V. A.; Rednic M.; Pratt S. T. Photodissociation of i-C_3_H_7_I within the A band and anisotropy-based decomposition of the translational energy distributions. J. Chem. Phys. 2009, 130, 134306 10.1063/1.3094321. 19355731

[ref17] Corrales M. E.; Loriot V.; Balerdi G.; González-Vázquez J.; de Nalda R.; Bañares L.; Zewail A. H. Structural dynamics effects on the ultrafast chemical bond cleavage of a photodissociation reaction. Phys. Chem. Chem. Phys. 2014, 16, 8812–8818. 10.1039/c3cp54677b. 24418888

[ref18] Atkinson R.; Baulch D. L.; Cox R. A.; R F Hampson J.; Kerr J. A.; Rossi M. J.; Troe J. Evaluated kinetic, photochemical and heterogeneous data for atmospheric chemistry: Supplement V. IUPAC subcommittee on gas kinetic data evaluation for atmospheric chemistry. J. Phys. Chem. Ref. Data 1997, 26, 521 10.1063/1.556011.

[ref19] Young C. J.; Hurley M. D.; Wallington T. J.; Mabury S. A. Atmospheric chemistry of 4:2 fluorotelomer iodide (n-C_4_F_9_CH_2_CH_2_I): Kinetics and products of photolysis and reaction with OH radicals and Cl atoms. J. Phys. Chem. A 2008, 112, 13542 10.1021/jp807322x. 19053571

[ref20] Boschi R. A.; Salahub D. R. The far ultra-violet spectra of some 1-iodoalkanes. Mol. Phys. 1972, 24, 289 10.1080/00268977200101451.

[ref21] Phillips D. L.; Myers A. B.; Valentini J. J. Investigation of solvation effects on short-time photodissociation dynamics of alkyl iodides. J. Chem. Phys. 1992, 96, 2039 10.1021/j100184a006.

[ref22] Warne E. M.; Downes-Ward B.; Woodhouse J.; Parkes M. A.; Bellshaw D.; Springate E.; Majchrzak P.; Zhang Y.; Karras G.; Wyatt A. S.; et al. Photodissociation dynamics of CH_3_I probed via multiphoton ionisation photoelectron spectroscopy. Phys. Chem. Chem. Phys. 2019, 21, 11142 10.1039/C9CP01477B. 31094379

[ref23] Downes-Ward B.; Warne E. M.; Woodhouse J.; Parkes M. A.; Springate E.; Pearcy P. A. J.; Zhang Y.; Karras G.; Wyatt A. S.; Chapman R. T.; Minns R. S. Photodissociation dynamics of methyl iodide across the A-band probed by femtosecond extreme ultraviolet photoelectron spectroscopy. J. Phys. B: Atom. Mol. Opt. Phys. 2021, 54, 134003 10.1088/1361-6455/ac08f3. 33146165

[ref24] Rubio-Lago L.; Garcıa-Vela A.; Arregui A.; Amaral G. A.; Bañares L. The photodissociation of CH_3_I in the red edge of the A-band: Comparison between slice imaging experiments and multisurface wave packet calculations. J. Chem. Phys. 2009, 131, 174309 10.1063/1.3257692. 19895014

[ref25] González M. G.; Rodrıguez J. D.; Rubio-Lago L.; Garcıa-Vela A.; Bañares L. Slice imaging and wave packet study of the photodissociation of CH_3_I in the blue edge of the A-band: evidence of reverse ^3^*Q*_0_ ←^1^*Q*_1_ non-adiabatic dynamics. Phys. Chem. Chem. Phys. 2011, 13, 16404–16415. 10.1039/c1cp21378d. 21847502

[ref26] Papadakis V.; Kitsopoulos T. N. Slice imaging and velocity mapping using a single field. Rev. Sci. Instrum. 2006, 77, 083101 10.1063/1.2222084.

[ref27] Rubio-Lago L.; Amaral G. A.; Oldani A. N.; Rodrıguez J. D.; González M. G.; Pino G. A.; Bañares L. Photodissociation of pyrrole–ammonia clusters by velocity map imaging: mechanism for the H-atom transfer reaction. Phys. Chem. Chem. Phys. 2011, 13, 1082–1091. 10.1039/C0CP01442G. 21076731

[ref28] Suits A. G. Invited Review Article: Photofragment imaging. Rev. Sci. Instrum. 2018, 89, 111101 10.1063/1.5045325. 30501356

[ref29] Rakitzis T. P.; Zare R. N. Photofragment angular momentum distributions in the molecular frame: Determination and interpretation. J. Chem. Phys. 1999, 110, 3341 10.1063/1.478200.

[ref30] Rakitzis T. P. Direct measurements of photofragment alignment from unnormalized Abel-inverted images. Chem. Phys. Lett. 2001, 342, 121–126. 10.1016/S0009-2614(01)00574-7.

[ref31] Recio P.; Cachón J.; Rubio-Lago L.; Chicharro D. V.; Zanchet A.; Limão-Vieira P.; Oliveira N.; Samartzis P. C.; Poullain S. M.; Bañares L. Imaging the Photodissociation Dynamics and Fragment Alignment of CH_2_BrI at 193 nm. J. Phys. Chem. A 2022, 126, 8404–8422. 10.1021/acs.jpca.2c05897. 36322967

[ref32] Orr-Ewing A. J.; Zare R. N. Orientation and Alignment of Reaction Products. Annu. Rev. Phys. Chem. 1994, 45, 315–366. 10.1146/annurev.pc.45.100194.001531.

[ref33] Samartzis P. C.; Bakker B. L. G.; Rakitzis T. P.; Parker D. H.; Kitsopoulos T. N. Spin-orbit branching ratios for the Cl atom photofragments following the excitation of Cl_2_ from 310 to 470 nm. J. Chem. Phys. 1999, 110, 5201 10.1063/1.478415.

[ref34] Eppink A. T. J. B.; Parker D. H. Energy partitioning following photodissociation of methyl iodide in the A band: A velocity mapping study. J. Chem. Phys. 1999, 110, 832 10.1063/1.478051.

[ref35] Kim Y. S.; Kang W. K.; Kim D.-C.; Jung K.-H. Photodissociation of tert-Butyl Iodide at 277 and 304 nm: Evidence for Direct and Indirect Dissociation in A-Band Photolysis of Alkyl Iodide. J. Phys. Chem. A 1997, 101, 7576 10.1021/jp970574z.

[ref36] Paterson C.; Godwin F.; Gorry P. Photofragmentation dynamics of C_2_H_5_I and CF_3_CH_2_I at 248 nm. Mol. Phys. 1987, 60, 729–747. 10.1080/00268978700100501.

[ref37] Godwin F.; Paterson C.; Gorry P. Photofragmentation dynamics of *n*-C_3_H_7_I and *i*-C_3_H_7_I at 248 nm. Mol. Phys. 1987, 61, 827–848. 10.1080/00268978700101501.

[ref38] Rakitzis T. P.; Kandel S. A.; Alexander A. J.; Kim Z. H.; Zare R. N. Measurements of Cl-atom photofragment angular momentum distributions in the photodissociation of Cl_2_ and ICl. J. Chem. Phys. 1999, 110, 3351 10.1063/1.478201.

